# Mining of Leaf Rust Resistance Genes Content in Egyptian Bread Wheat Collection

**DOI:** 10.3390/plants10071378

**Published:** 2021-07-05

**Authors:** Mohamed A. M. Atia, Eman A. El-Khateeb, Reem M. Abd El-Maksoud, Mohamed A. Abou-Zeid, Arwa Salah, Amal M. E. Abdel-Hamid

**Affiliations:** 1 Molecular Genetics and Genome Mapping Laboratory, Genome Mapping Department, Agricultural Genetic Engineering Research Institute (AGERI), Agricultural Research Center (ARC), Giza 12619, Egypt; asalah@matia.org; 2 Department of Botany, Faculty of Science, Tanta University, Tanta 31527, Egypt; eman.elkhateb@science.tanta.edu.eg; 3 Department of Nucleic Acid & Protein Structure, Agricultural Genetic Engineering Research Institute (AGERI), Agricultural Research Center (ARC), Giza 12619, Egypt; reem.mohsen@ageri.sci.eg; 4 Wheat Disease Research Department, Plant Pathology Research Institute, Agricultural Research Center (ARC), Giza 12619, Egypt; mabouzeid@matia.org; 5 Department of Biological and Geological Sciences, Faculty of Education, Ain Shams University, Roxy, Cairo 11341, Egypt; amelmohamed@edu.asu.edu.eg

**Keywords:** *Triticum aestivum*, *Lr* resistance gene, *Puccinia triticina*, resistance genes, molecular markers

## Abstract

Wheat is a major nutritional cereal crop that has economic and strategic value worldwide. The sustainability of this extraordinary crop is facing critical challenges globally, particularly leaf rust disease, which causes endless problems for wheat farmers and countries and negatively affects humanity’s food security. Developing effective marker-assisted selection programs for leaf rust resistance in wheat mainly depends on the availability of deep mining of resistance genes within the germplasm collections. This is the first study that evaluated the leaf rust resistance of 50 Egyptian wheat varieties at the adult plant stage for two successive seasons and identified the absence/presence of 28 leaf rust resistance (*Lr*) genes within the studied wheat collection. The field evaluation results indicated that most of these varieties demonstrated high to moderate leaf rust resistance levels except Gemmeiza 1, Gemmeiza 9, Giza162, Giza 163, Giza 164, Giza 165, Sids 1, Sids 2, Sids 3, Sakha 62, Sakha 69, Sohag 3 and Bany Swif 4, which showed fast rusting behavior. On the other hand, out of these 28 *Lr* genes tested against the wheat collection, 21 *Lr* genes were successfully identified. Out of 15 *Lr* genes reported conferring the adult plant resistant or slow rusting behavior in wheat, only five genes (*Lr13*, *Lr22a*, *Lr34*, *Lr37*, and *Lr67)* were detected within the Egyptian collection. Remarkedly, the genes *Lr13*, *Lr19*, *Lr20*, *Lr22a*, *Lr28*, *Lr29*, *Lr32*, *Lr34*, *Lr36*, *Lr47*, and *Lr60*, were found to be the most predominant *Lr* genes across the 50 Egyptian wheat varieties. The molecular phylogeny results also inferred the same classification of field evaluation, through grouping genotypes characterized by high to moderate leaf rust resistance in one cluster while being highly susceptible in a separate cluster, with few exceptions.

## 1. Introduction

*Triticum aestivum* L. (bread wheat) is an essential grain worldwide, including Egypt. It provides humanity their protein requirements [1,2]. In developing countries, the requirement for wheat grains increased every year and is predicted to reach 60% by 2050 [3]. The agriculture of most regions has experienced adverse effects from climate change, which constitutes the main cause of biotic and abiotic stresses. Climate change (drought, high temperatures, pests, floods, storm, and disease epidemics) greatly affects crop production globally [4]. Generally, wheat plays a fateful role in global food security, especially in Egypt’s food economy, from production and consumption perspectives [5].

Rusts and powdery mildew are wheat pathogens that cause a vital decrease in wheat production [5]. In wheat, three species of wheat rust were found: leaf or brown rust (*Puccinia triticina*), stem or black rust (*Puccinia graminis* f. sp. *tritici*) and stripe or yellow rust (*Puccinia striiformis* f. sp. *tritici*) [6,7]. Rusts are caused by Puccinia spp., considered to be the most significant wheat disease and representing a big concern for wheat breeders and farmers [8]. It leads to tremendous strike yield losses annually to wheat crops worldwide [9,10].

The worldwide plant production is decreased by at least 10% because of diseases and pests [11]. Among wheat rust diseases, the disease of leaf rust is still the number one widespread and destructive wheat disease, and it is the most important biotic stress that limited the productivity of bread wheat in Egypt and worldwide [12]. It has caused significant losses in grain yield, which have reached 23% [13], and the losses in epidemic seasons have reached up to 50% [14]. Leaf rust has the capability of adapting to varied climatological conditions; therefore, it is one of the most devastating diseases of wheat worldwide [15].

In general, wheat leaf rust occurs more frequently and spreads widely disease compared to the other two wheat rusts (stem and stripe rust) and is more prevalent globally [16]. The pathogen is able to spread thousands of kilometers via wind [9] and acclimate to diverse temperatures [17]. It causes severe damage to yields worldwide due to decreases grain quality and quantity (yield production) by greater than 50% in susceptible varieties under the pathogens’ favorable environmental conditions [18].

Fungicides are the most favored method by breeders to fight fungal diseases, but they are expensive and can harm the environment [19]. Genetic resistance breeding is an efficient, economical and environmentally friendly way to relieve pathogen damage [20], saving time and effort compared to traditional methods [21].

Genetic resistance to leaf rust is generally classified into two forms: adult-plant resistance (APR) and seedling resistance (ASR) [8,22]. Seedling resistance (ASR) is monogenic, usually expressed at all growth stages, controlled by a single (major effect) gene, and hypersensitive. On the contrary, adult plant resistance (APR) is polygenic, typically best expressed in adult plants controlled by multiple (minor effect) genes, and non-hypersensitive, slow rusting [23]. Additionally, it can distinguish the resistance to leaf rust into two types, qualitative conferred by single resistance genes and quantitative resistance, facilitated by multiple genes [24].

One of the efficient methods used for leaf rust disease control is host-genetic resistance. It is an economical, environmentally safe approach and eliminates the use of synthetic fungicides. Race-specific genes for resistance allow enough protection against only a few pathotypes of the pathogen and interact according to the gene-for-gene theory [25]. Additionally, this method aimed to obtain high-yielding varieties characterized by high levels of resistance to major diseases, especially wheat leaf rust [22].

Recently, it has become possible to directly identify the *Lr* genes using specific primers targeting the *Lr* genes itself or by using linked molecular markers, such as simple sequence repeat (SSR), sequence-tagged site (STS), sequence-characterized amplified regions (SCAR) and cleaved amplified polymorphic sequences (CAPS) [26]. Molecular markers, particularly PCR-based markers, proved their efficient ability to identify resistance genes in varieties and to combine them accurately toward selecting lines with suitable gene pool combinations [27]. Thus, the introgression of the leaf rust resistance genes (*Lr* genes) into wheat varieties using molecular markers (specific or linked) is the superior approach of their safeguarding in terms of environmental protection [26].

Most leaf rust (*Lr*) resistance genes are conferred as major, seedling, or race-specific and follow up the gene-for-gene theory [28]; unfortunately, when new races of the pathogen appear, they lose their efficacy [29]. Presently, there is a crucial need for breeding programs focusing on producing varieties with adult plant resistance (APR) [19]. Likewise, wheat varieties differ in disease resistance depending on which *Lr* genes they carry, or, in other words, their *Lr* gene pool [26].

Nowadays, more than 100 leaf rust resistance (*Lr*) genes/alleles have been characterized, recognized, and described in seedling and adult plant resistance to leaf rust in wheat and its relatives [7,30]. The majority of them are originated from hexaploid bread wheat or wild grass species related to wheat, whereas a limited number have been identified and characterized in tetraploid durum wheat [31]. A few race non-specific *Lr* genes, including *Lr*34 and *Lr*67, have been found, mainly at the adult stage, conferring resistance to multiple pathogen species [32].

Unfortunately, limited information is known about the *Lr* genes content within Egyptian wheat varieties [33,34]. Therefore, this study evaluates the disease resistance level of the 50 Egyptian wheat varieties against leaf rust at the adult plant stage under open field conditions (two successive seasons) and identifies varieties with slow-rusting characteristics. Additionally, we define the leaf rust resistance genes’ content in the studied Egyptian wheat varieties/collection to act as a core base for successful and effective breeding programs for leaf rust resistance.

## 2. Results

### 2.1. Field Evaluation of Leaf Rust Resistance

The tested wheat varieties were evaluated under field conditions for their final rust severity (FRS), area under the disease progress curve (AUDPC) and rate of leaf rust disease increase (r-value) traits. According to the obtained records during the two growing seasons and based on recorded values of the final rust severity (%), the tested varieties have been clustered under three main groups: (a) varieties with moderate to high levels of resistance (Mabrok, Sakha 95, Montana, Sohag 5, Misr 2 and Giza 168) (FRS ranged between 0.33 and 2.32%), (b) varieties with intermediate to low levels of resistance (Giza 139, Giza 144, Giza 155, Giza 156, Giza 157, Giza 160, Giza 167, Giza 171, Sakha 8, Sakha 61, Sakha 88, Sakha 92, Sakha 94, Sids 8, Sids 5, Sids 6, Sids 7, Gemmeiza 7, Sids 14, Romana, Hendy 62, Nubaria 1, Gemmeiza 5, Gemmeiza 3, Gemmeiza 11, Gemmeiza 12, BanySwif 1, BanySwif 5, BanySwif 6, BanySwif 7 and Sohag 4) (with FRS less than 30%), and (c) highly susceptible varieties (Gemmeiza 1, Gemmeiza 9, Giza162, Giza 163, Giza 164, Giza 165, Sids 1, Sids 2, Sids 3, Sakha 62, Sakha 69, Sohag 3 and BanySwif 4) (with FRS more than 60%); the data are presented in Table 1.

Additionally, the area under disease progress curve (AUDPC) parameter we used as a convenient and reliable criterion to describe the performance of durable resistance in any wheat variety. It combines the infection period and the level of infection and uses this combination to estimate (AUDPC) values for the tested varieties. In general, the values of (AUDPC) were found to be higher in the second season than in the first season. Based on the AUDPC records of the two seasons, the tested varieties could be divided into the following three groups. The first group included wheat varieties with the lowest AUDPC (less than 100); these cultivars were designated as the resistance varieties or partial resistance. This group includes six wheat varieties (Mabrok, Sakha 95, Montana, Sohag 5, Misr 2 and Giza 168) and their AUDPC values ranged from 5 to 63. The second group included the varieties with intermediate and low AUDPC (ranged from 101 to 390), and these varieties revealed the lowest levels of adult plant resistance to leaf rust infection under field conditions. This group includes varieties Giza 139, Giza 144, Giza 155, Giza 156, Giza 157, Giza 160, Giza 167, Giza 171, Sakha 8, Sakha 61, Sakha 88, Sakha 92, Sakha 94, Sids 8, Sids 5, Sids 6, Sids 7, Gemmeiza 7, Sids 14, Romana, Hendy 62, Nubaria 1, Gemmeiza 5, Gemmeiza 3, Gemmeiza 11, Gemmeiza 12, BanySwif 1, BanySwif 5, BanySwif 6, BanySwif 7 and Sohag 4.

The third group included the wheat varieties that showed the highest estimated AUDPC levels; the varieties in this group were classified as the highly susceptible or fast leaf rusting Egyptian wheat varieties (Gemmeiza 1, Gemmeiza 9, Giza162, Giza 163, Giza 164, Giza 165, Sids 1, Sids 2, Sids 3, Sakha 62, Sakha 69, Sohag 3 and BanySwif 4) with AUDPC values ranged from 610 to 111.

Rusting progression in tested wheat varieties was estimated based on the “rate of leaf rust increase lower rates (r-value)” parameter, and the 50 varieties were classified into three categories based on their r-values: (a) varieties exhibited almost complete resistance (their r-value ranged from 0.030 to 0.080); (b) varieties with slow leaf rusting rates, which exhibited intermediate levels of r-value (ranged from 0.090 to 0.180); and (c) fast rusting or highly susceptible wheat varieties, where the r-values reached the maximum levels (ranged from 0.200 to 0.218).

The analysis of variance results showed that the mean squares of the 50 wheat genotypes were highly significant for all the studied disease parameters during the two growing seasons as shown in Table 2. These results showed that all the genotypes differed in their response to leaf rust disease.

### 2.2. Screening for Leaf Rust Resistance Genes within Egyptian Wheat Collection

Fifty Egyptian wheat varieties were characterized using 28 *Lr* genes (*Lr1, Lr9, Lr10, Lr13, Lr19, Lr20, Lr21, Lr22a, Lr24, Lr25, Lr26, Lr27, Lr28, Lr29, Lr32, Lr34, Lr35, Lr36, Lr37, Lr39, Lr46, Lr47, Lr48, Lr50, Lr52, Lr60, Lr63*, and *Lr67*) as molecular markers controlling the leaf rust resistance in the selected Egyptian wheat varieties. The primer names, sequences, annealing temperature and size of the amplified fragment for *Lr* genes markers used in this study are shown in Table 3.

The molecular characterization results revealed that the 28 *Lr* tested genes yielded a total number of 34 scorable bands/amplicons. Out of the 28 *Lr* genes, nine *Lr* genes were found to have more than one allele (*Lr13, Lr20, Lr24, Lr27, Lr37, Lr39, Lr47, Lr63,* and *Lr*67). Interestingly, six *Lr* resistance genes (*Lr22a, Lr28, Lr29, Lr32, Lr34,* and *Lr47-1*) were found to be represented in all the studied varieties or, in other words, revealed a monomorphic pattern between all varieties. Notably, the *Lr10* resistance gene was considered the only unique positive marker gene since it showed a scorable band only in the BanySwif 5 variety, while it was absent in all of the other 49 studied varieties (Table 4).

In terms of polymorphism levels, all primers showed a high percentage of polymorphism, except *Lr*22a, *Lr*28, *Lr*29, *Lr*32, and *Lr*34, which showed absolute monomorphism. A narrow range of the expected heterozygosity values (H) were observed; the values were ranged between 0.0 to 0.5. A total of 9 out of the 28 primers showed very close and similar values near to 0.5. Moreover, the polymorphism information content (PIC) also exhibited the same range with values ranged from 0.0 to 0.5. The effective multiplex ratio values were more varied comparing to H and PIC; their values ranged from 0.02 to 1.94. On the other hand, the marker index and mean heterozygosity values were very low (ranging from 0.0000 to 0.00569). The discriminating power values ranged from 0.0 to 1.0. Additionally, the resolving power values were ranged between 0.0 and 2.12. The primer *Lr*27 showed the best resolving power among all the used *Lr* primers (Table 5).

**Figure 1:**
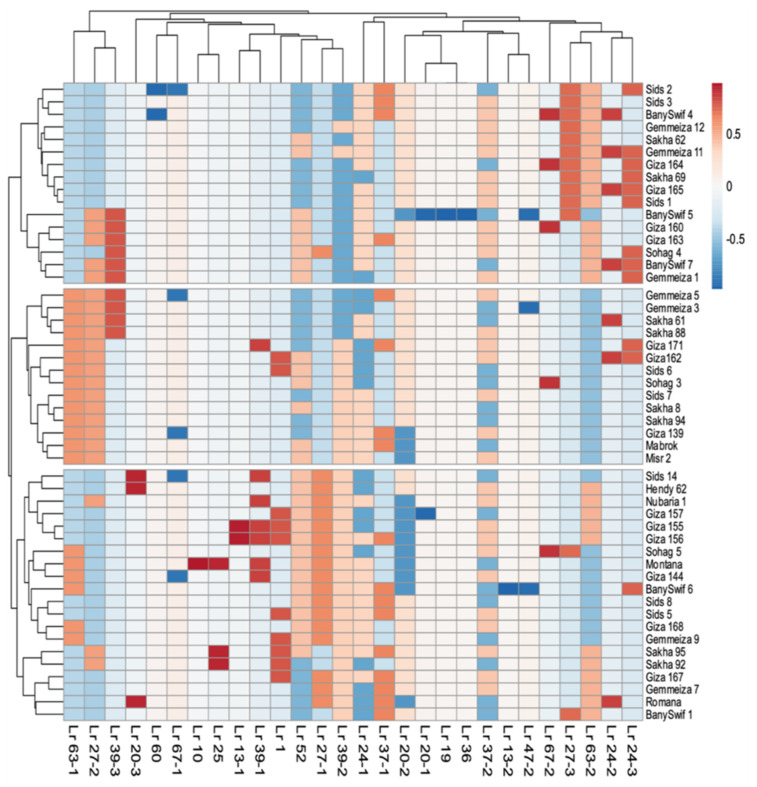
Two-dimensional heatmap showing the clustering of the 50 wheat varieties based on the presence of *Lr* genes’ and varieties’ performance revealed grouping into three distinct groups. Rows represent the 50 wheat genotypes and columns represent the *Lr* genes/alleles.

On the other side, the two-dimensional heatmap visualization of the interaction between the presence of *Lr* genes and varieties performance revealed grouping of the 50 wheat varieties into three distinct groups (Figure 1).

**Figure 2:**
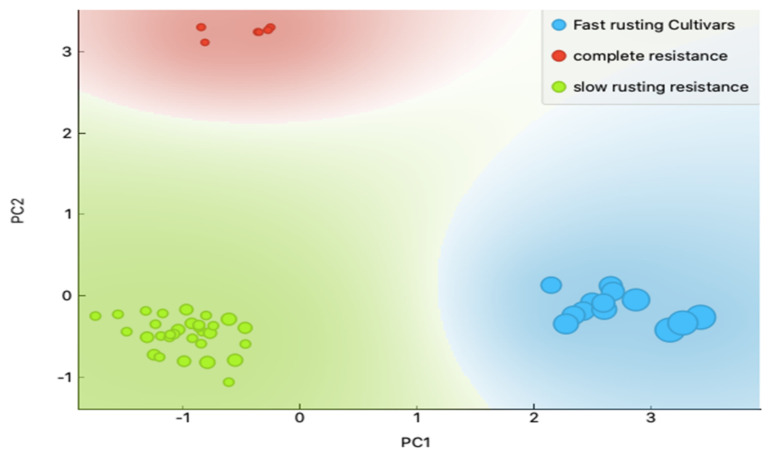
Principal component analysis (PCA) based on the *Lr* genes’ scoring data. The figure demonstrates a sharp clustering into three distinctive groups (green: slow rusting resistance genotypes; red: complete resistance genotypes; blue: slow rusting resistance genotypes).

For principal component analysis (PCA), a scatter plotting of principal component 1 (PC1) plotted against principal component 2 (PC2) successfully separated the 50 wheat varieties into three sharp groups: (1) complete resistance varieties (red; Mabrok, Sakha 95, Montana, Sohag 5, Misr 2 and Giza 168), (2) fast rusting varieties (blue; Gemmeiza 1, 9, Giza162, 163, 164, 165, Sids 1, 2, 3, Sakha 62, 69, Sohag 3 and BanySwif 4), and 3) slow rusting resistance (green; Nubaria 1, Giza 139, 144, 155, 156, 157, 160, 167, 171, Sakha 8, 61, 88, 92, 94, Sids 5, 6, 7, 8, 14, Gemmeiza 3, 5, 7, 11, 12, BanySwif 1, 5, 6, 7, Romana, Hendy 62 and Sohag 4) (Figure 2).

**Figure 3:**
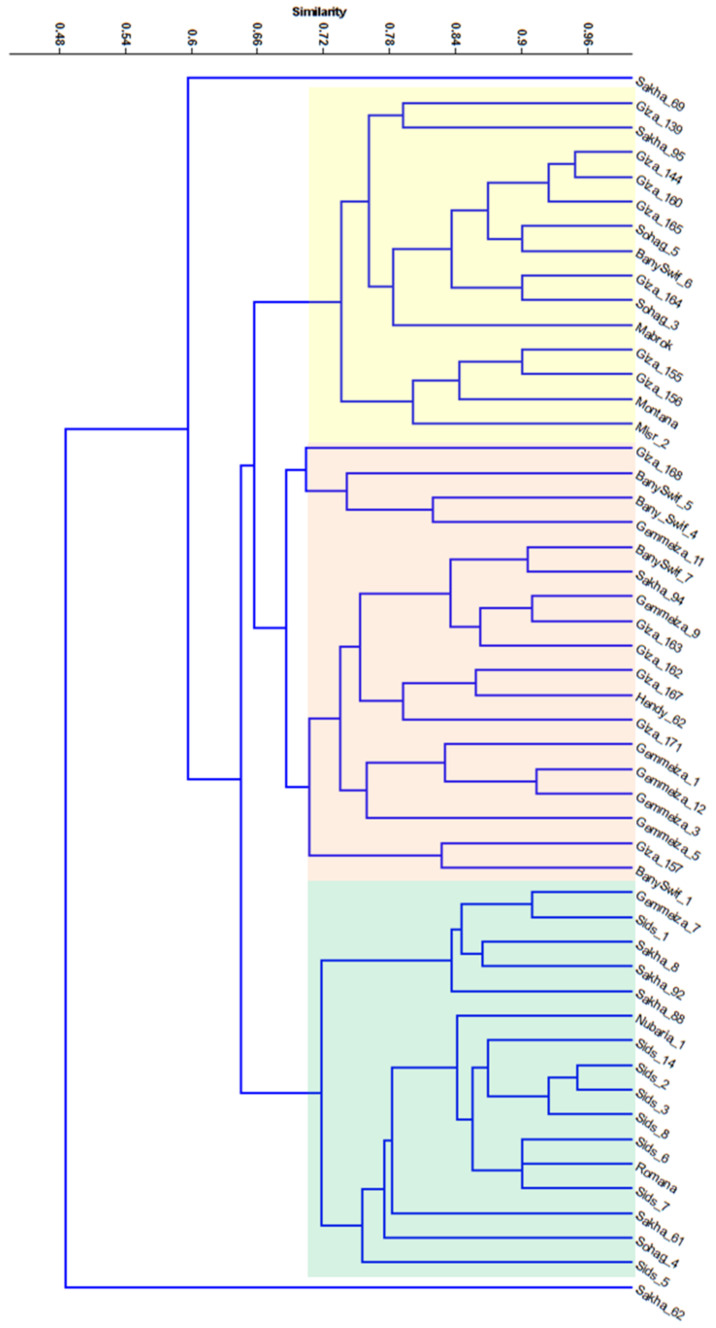
Phylogenetic tree showing the similarity among 50 wheat varieties based on Jaccard’s similarity analysis of 28 *Lr* genes markers.

Dendrograms based on UPGMA analysis of *L r* markers data were constructed for the 50 wheat varieties (Figure 3). The dendrogram comprised two main clusters. The first cluster comprised only the Sakha 62 variety, while the second cluster comprised the other 49 wheat varieties. Particularly, the second cluster was subdivided into two main sub-clusters; the first sub-cluster comprised only the Sakha 69 variety, while the second sub-cluster was subdivided into three separate groups (Figure 3).

## 3. Discussion

Rusts are one of the most destructive biotic stress in wheat and represent significant production constraints to wheat crop productivity worldwide. It has caused significant yield losses (about 60%) and diminished the quality of wheat grains. Three distinct types of rust diseases attack wheat: leaf rust (LR), yellow rust (YR), and stem rust (SR). These rusts are caused by certain pathogen species, consequently having many pathotypes that parasitize certain wheat varieties. Leaf rust disease caused by the fungus *Puccinia triticina* (Eriks) is the most common bread wheat rust, causing significantly massive yield losses in wheat crops worldwide [57]. One of the essential steps in which molecular marker techniques are used in developing efficient wheat breeding programs for rust resistance is determining and characterizing the wheat genotypes for their carrying of leaf rust resistance genes. 

During the last decade, the dramatic development of molecular marker techniques and gene identification has facilitated the establishment of effective marker-assisted selection (MAS) systems, particularly towards wheat breeding for leaf rust resistance. These MAS systems were successfully established due to PCR-based markers’ availability for almost 80 designated leaf resistance genes/alleles [23]. Remarkably, few studies have been released during the last decades describing the wheat germplasm that carries *Lr* genes, especially in Egypt. This apparent lack of knowledge might be attributed to the giant genome of wheat and the existence of various pathotypes (races) that attack only certain varieties of wheat [58].

Therefore, this study aimed to evaluate 50 Egyptian wheat varieties’ performance under open field conditions against leaf rust disease for two growing seasons (2018/2019 and 2019/2020) at the adult plant stage. The area under the disease progress curve (AUDPC) was used as the most reliable and convenient estimator to accurately measure the amount of rust infection. Based on the AUDPC, the evaluation results of these 50 varieties indicated that most of these varieties exhibited high to moderate leaf rust resistance, except varieties Gemmeiza 1, Gemmeiza 9, Giza162, Giza 163, Giza 164, Giza 165, Sids 1, Sids 2, Sids 3, Sakha 62, Sakha 69, Sohag 3, and BanySwif 4, which exhibited highly susceptible or fast leaf rusting behavior. Similar results were reported by Fahmi et al. (2015) [59], and Pathan and Park (2006) [60], who found that the Giza 163, Giza 164, Sids 1, and Sakha 69 wheat varieties were highly susceptible, compared to slow-rusting cultivars. The durability of resistance is supported by the diversity of resistant genes.

On the other side, we characterized these 50 varieties for their leaf rust resistance genes (28 *Lr* genes). Out of these 28 *Lr* genes, 21 *Lr* genes (*Lr1*, *Lr10*, *Lr13*, *Lr19*, *Lr20*, *Lr22a*, *Lr24*, *Lr25*, *Lr27*, *Lr28*, *Lr29*, *Lr32*, *Lr34*, *Lr36*, *Lr37*, *Lr39*, *Lr47*, *Lr52*, *Lr60*, *Lr63*, *Lr67*) were successfully identified. Out of 15 *Lr* genes reported conferring the adult plant resistant or slow rusting behavior in wheat [16,61,62], only five genes (*Lr13*, *Lr22a*, *Lr34*, *Lr37* and *Lr67)* were observed within the 50 Egyptian wheat varieties. The genes *Lr13*, *Lr19*, *Lr20*, *Lr22a*, *Lr28*, *Lr29*, *Lr32*, *Lr34*, *Lr36*, *Lr47*, and *Lr60* were the most predominant leaf rust resistance genes recognized across the 50 Egyptian wheat varieties. Among these genes, the *Lr13* gene was previously reported as the most broadly distributed *Lr* gene worldwide [63]. Moreover, it has been described that across European wheat genotypes, 58% of these tested genotypes were found to carry the *Lr13* gene alone or in combination with other resistance genes [60,64]. Moreover, Australian wheat genotypes were also found to contain this gene singly or combined with other race-specific genes [65].

Regarding *Lr34*, as expected, we identified this gene in almost all test Egyptian varieties, which is in good accordance with previous reports of Singh and Rajaram (1992) [66], Imbaby et al., (2014) [67] and Fahmi et al. (2015) [59]. For *Lr37*, which has been reported to mainly confer the adult plant’s resistance rather than in seedlings, it was found to be represented in about 60% of the tested Egyptian wheat genotypes. These results agreed with previous reports of Imbaby et al. (2014) [47] on Egyptian germplasm and Singh and Rajaram (2002) [65] on Western European germplasm. Meanwhile, *Lr19* and *Lr24*, which were reported to be genetically linked to stem rust resistance genes *Sr25* and *Sr24*, were found to be represented in about 98% and 66% of the tested wheat genotypes, respectively.

For *Lr28* and *Lr29*, it was previously observed in 5 and 10 Egyptian wheat varieties, respectively [5]. They also found that these varieties carried the *Lr25* and *Lr67* genes that might explain their higher degree of resistance. This finding was in complete agreement with our obtained results which confer that the *Lr28* and *Lr29* genes were represented in all the tested Egyptian wheat genotypes.

From another perspective, the molecular phylogeny analysis of the Egyptian wheat collection revealed accordance classification with the field evaluation of leaf rust resistance results with a little dissimilarity. The dendrogram gathered the genotypes characterized by high to moderate leaf rust resistance in one cluster while keeping those who exhibited highly susceptible or fast leaf rusting performance in a separate cluster, with a few exceptions.

The marker-assisted selection approaches grant the opportunity to select lines with desirable traits based on their genetic constituents rather than phenotypic performance, especially those combining several genes in a single genotype. With the guidance of molecular marker techniques, the pyramiding breeding of *Lr* genes (even those functional at the seedling and/or adult plant stages) is expected to facilitate the designing of efficient breeding programs for durable resistance against this pandemic wheat disease. Undoubtedly, the resistance mechanisms against leaf rust are still poorly understood, but the information gained from resistance genes that are found in many varieties can help breeders to develop resistant varieties [67]. This could be the most ecological and economical solution to manage wheat rust disease [8]; additionally, the resistance durability seems to be dramatically improved when Lr resistance genes are combined [68], whereby when more LR genes are accumulated in a variety, the combined effects of these genes give this variety a large base to resistance to disease [8].

Therefore, molecular markers can be used effectively to confirm the existence of desired *Lr* resistance genes within the genetic background of certain wheat varieties and, consequently, to choose the most appropriate parents for efficient breeding programs.

## 4. Materials and Methods

### 4.1. Plant Material

Fifty varieties of Egyptian wheat were tested for their response to leaf rust. The wheat varieties were provided by the Wheat Diseases Research Department, Plant Pathology Research Institute, Agricultural Research Center (ARC), Giza, Egypt. The wheat varieties include BanySwif (1, 4, 5, 6, 7), Gemmeiza (1, 3, 5, 7, 9, 11, 12), Giza (139, 144, 155, 156, 157, 160, 162, 163, 164, 165, 167, 168, 171), Sakha (8, 61, 62, 69, 88, 92, 94, 95), Sids (1, 2, 3, 5, 6, 7, 8, 14), Sohag (3, 4, 5), Hendy 62, Mabrok, Misr 2, Montana, Nubaria 1, and Romana (Table 6). These varieties were tested for their leaf rust resistance under open field conditions at the adult plant stage. The experiments of the current study were carried out under field conditions at Sids Agricultural Research Station during two successive growing seasons—2018/19 and 2019/20.

### 4.2. Inoculation and Disease Assessment

Artificial inoculation of 75-day-old plants was carried out to ensure a threshold of infection. This was carried out in the evening with a mixture of freshly collected urediospores of the prevalent leaf rust races and talcum powder at a rate of 1: 20 (*v*/*v*) using baby cyclone to assure rapid, uniform deposition of spores onto all plants [69].

Leaf rust severities were determined using the modified Cobbs scale from 0 to 100% [70], as the percentage of leaf surface area covered by the fungus structure.

Disease severity was assessed using two epidemiological parameters—the final rust severity (FRS%) and the area under disease progress curve (AUDPC)—these scores were used to calculate the area under the disease progress curve (AUDPC) as described by Roelfs et al., (1992) [70]. The final rust severity was calculated for each cultivar as follows, which was expressed as a percentage of leaf area covered with leaf rust (0% to 100%), recorded according to the modified Cobbs scale [71].

Final rust severity (FRS%) was also recorded for each of the tested varieties as the disease severity (%) when the highly susceptible (check) variety was severely rusted, and the disease rate reached its highest or final level of severity [72]. The area under the disease progress curve (AUDPC) was calculated using the formula suggested by Pandey et al. (1989) [73].

AUDPC = D [(Y_1_ + Y_k_) + (Y_2_ + Y_3_ + …… + Y_k−1_)]

where:

D = days between two consecutive records (time intervals)

Y_1_ + Y_k_ = sum of the first and last disease scores.

Y_2_ + Y_3_ + …….. + Y_k−1_ = sum of all in between disease scores.

The rate of leaf rust disease increase (r-value), as a function of times, was also estimated, according to Van der Plank (1963) [74].

A combined analysis of variance over the two seasons was carried out (Table 5). The importance of difference among the studied varieties was tested by carefully studying variance (ANOVA) which was carried out for each year separately. The test was as organized and listed by Snedecor and Cochran (1967) [75]. Mean comparisons for numbers that change were made among genotypes using the least big differences (LSD at 5%) tests (Table 6).

### 4.3. Molecular Analysis

#### 4.3.1. DNA Extraction

Genomic DNA of the 50 varieties was isolated from green leaves, using an i-genomic Plant DNA Extraction Mini Kit (iNtRON, Seongnam, Korea), used according to the manufacturer’s instructions. The isolated DNA was measured using a NanoDrop 2000 spectrophotometer (Thermo Scientific, Bremen, Germany) to calculate the concentration and purity; each sample of total DNA was loaded into 1% agarose gel to test the integrity of DNA.

#### 4.3.2. Molecular Detection of *Lr* Genes

Specific primers were used to verify the presence of 28 *Lr* genes in 50 varieties (Table 2). All primers were obtained from previous studies except the *Lr*22a, and *Lr*48 primers were designed based on two sequences of leaf rust resistance genes available on the National Center for Biotechnology Information (NCBI) database.

#### 4.3.3. PCR amplification and Gel Analysis

The PCR reaction mixture (25 μL) contained 30 ng DNA template, 10 pmol of forward primer, 10 pmol of reverse primer, 0.1 U of Go-Taq Flexi polymerase (Promega), 25 mM of MgCl_2_, 2 mM dNTPs, and 5 × PCR buffer. The reaction conditions of amplification were as follows: initial denaturation at (94 °C for 4 min), followed by 40 cycles at (94 °C for 1 min; the annealing temperature was adjusted according to each primer for 1 min, 72 °C for 2 min), and final extension (72 °C for 5 min), then held at 4 °C [42]. The amplification of PCR products was performed in a GeneAmp^®^ PCR System 9700 (Applied Biosystems, Forster City, CA, USA). The sequences of the used primers and expected fragment sizes are listed in Table 2.

The products amplified were separated by electrophoresis in 2% agarose gel with 0.5 × TBE buffer at 100 volts for 45 min and stained with ethidium bromide (10 mg/mL). The bands were visualized using UV light transilluminator followed by being photographed with a gel documentation system (Molecular Imager^®^ Gel Doc™ XR + System with Image Lab™ Software, Bio-Rad Laboratories, Hercules, CA, USA). GeneRuler100bp DNA Ladder Plus (Fermentas, Opelstrasse 9, Germany) was used as a standard molecular weight marker.

### 4.4. Data Analysis

For molecular data analysis, the generated/amplified bands were scored visually. To reduce errors, only the clear and distinguishable bands were scored. The bands were scored as present (1) or absent (0) to create the binary dataset [76]. The polymorphism percentage was calculated by dividing the number of amplified polymorphic bands by the total number of amplified bands by the same primer or primer combination. A similarity matrix was constructed to measure genetic distances between pairs of plants; these distances were estimated between all possible pairs [77]. The pairwise comparisons were made between the 50 wheat genotypes based on the Jaccard similarity coefficient [78]. The genetic similarity estimate (GS) between each pair of genotypes was calculated using the expression GS = a/(n−d), in which a is the number of positive coincidences, n is the total number of fragments, and d is the number of negative coincidences. The genetic distances (GD) between pairs of plants were estimated by GD = 1 − GS.

A dendrogram was generated by cluster analysis using the unweighted pair group method of the arithmetic averages (UPGMA) for all different marker systems using Past Software [79].

The efficiency of the characterized *Lr* genes/primers was determined by calculating the following parameters: expected heterozygosity (H = 1 − Σ pi^2^ according to Liu, 1998) [80], polymorphism information content (PIC = 1 − Σ pi^2^ − ΣΣ pi^2^ pj^2^ according to Botstein et al., 1980) [81], effective multiplex ratio (E = n β according to Powell et al., 1996) [82], Marker Index (MI = E Hav according to Powell et al., 1996) [82], mean heterozygosity (Hav = ΣH_n_/n_p_ according to Powell et al., 1996) [82], discriminating power (D = 1 − C according to Tessier et al., 1999) [83], resolving power (R = ΣI_b_ according to Prevost and Wilkinson, 1999) [84]. Finally, based on *Lr* scoring data combined with disease assessment records, hierarchical clustering and principal component analysis (PCA) were developed. The heatmap and PCA were drawn with aid of ClustVis. tool [85,86] and JavaScript script language [87].

## 5. Conclusions

The mining, characterization, and distribution of *Lr* genes within certain wheat genotypes/collections are crucial for developing new wheat-resistant genotypes. Gene pyramiding of *Lr* genes with the aid of molecular markers is necessary for ensuring the long-term sustainability of leaf rust resistance in Egyptian wheat varieties. In this study, 50 Egyptian wheat varieties were evaluated for their leaf rust resistance level at the adult plant stage for two successive seasons. The evaluation results indicated that most of the Egyptian wheat collection (37 out of 50 varieties) demonstrated high to moderate leaf rust resistance levels. Additionally, out of 28 *Lr* genes screened within the wheat collection, 21 *Lr* genes were successfully observed. Distinctly, 11 *Lr* genes (*Lr13, Lr19, Lr20, Lr22a, Lr28, Lr29, Lr32, Lr34, Lr36, Lr47,* and *Lr60*) were characterized as the most predominant *Lr* genes within the 50 Egyptian wheat varieties. Ultimately, our findings can act as a fundamental base for successful and efficient breeding programs for leaf rust resistance in an Egyptian wheat collection.

## Figures and Tables

**Table 1:** Final leaf rust severity (%), area under disease progress curve (AUDPC) and rate of leaf rust disease increase (r-value) in Sids location during 2018/19 and 2019/20 growing season.

Code	Varieties	FRS		AUDPC		r-Value	
		18/19	19/20	18/19	19/20	18/19	19/20
1	Mabrok	0.33	1.67	5.00	40.00	0.050	0.060
2	Sakha 95	1.67	2.00	2.50	63.00	0.060	0.050
3	Montana	0.67	0.67	23.50	70.50	0.080	0.080
4	Sohag 5	1.67	1.00	37.50	5.00	0.030	0.030
5	Misr 2	1.00	0.67	21.50	33.50	0.030	0.070
6	Giza 168	1.67	2.32	22.50	24.50	0.000	0.000
7	Nubaria 1	11.67	13.00	103.00	119.50	0.180	0.180
8	Giza 144	18.33	26.67	370.00	382.00	0.120	0.120
9	Giza 155	7.33	8.67	162.50	89.50	0.140	0.150
10	Giza 156	7.00	8.33	157.50	169.50	0.130	0.140
11	Giza 157	25.67	26.00	368.50	310.00	0.120	0.120
12	Giza 160	18.00	22.33	261.50	259.00	0.120	0.120
13	Giza 167	3.00	9.00	210.00	179.00	0.110	0.110
14	Giza 171	22.00	26.33	310.00	317.50	0.120	0.130
15	Sakha 8	8.33	8.00	180.00	187.50	0.110	0.120
16	Sakha 61	15.00	15.00	101.50	131.50	0.130	0.150
17	Sakha 88	11.00	7.00	102.50	109.00	0.090	0.090
18	Sakha 92	4.00	6.33	105.00	100.50	0.090	0.080
19	Sakha 94	9.00	13.00	106.50	106.50	0.090	0.090
20	Sids 8	4.00	4.00	115.00	132.50	0.110	0.120
21	Sids 5	5.00	5.00	108.50	137.50	0.140	0.140
22	Sids 6	10.67	18.67	140.00	171.50	0.120	0.130
23	Sids 7	10.00	21.67	137.50	156.00	0.110	0.130
24	Gemmeiza 7	22.33	22.00	278.00	232.50	0.090	0.080
25	Sids 14	11.00	11.67	158.00	194.50	0.120	0.140
26	Romana	11.33	12.00	102.50	109.50	0.090	0.090
27	Hendy 62	22.33	17.00	122.33	106.50	0.110	0.110
28	Giza 139	28.33	30.00	334.00	390.00	0.174	0.169
29	Gemmeiza 5	7.33	15.00	117.50	194.50	0.140	0.140
30	Gemmeiza 3	10.00	21.67	274.50	294.00	0.130	0.150
31	Gemmeiza 11	8.67	7.00	275.00	324.50	0.130	0.130
32	Gemmeiza 12	8.00	7.00	109.50	194.50	0.110	0.120
33	BanySwif 1	22.33	17.00	260.50	296.00	0.080	0.090
34	BanySwif 5	20.33	19.00	294.50	278.00	0.112	0.102
35	BanySwif 6	30.00	19.00	311.00	345.00	0.104	0.114
36	BanySwif 7	22.67	22.33	315.00	330.50	0.104	0.106
37	Sohag 4	22.33	22.00	343.00	305.50	0.126	0.105
38	Gemmeiza 1	61.67	65.00	646.50	666.00	0.215	0.218
39	Gemmeiza 9	75.00	60.00	708.50	701.00	0.200	0.218
40	Giza162	65.00	65.00	760.00	811.50	0.213	0.215
41	Giza 163	60.00	61.67	698.50	729.50	0.216	0.226
42	Giza 164	70.00	65.00	905.00	996.50	0.213	0.250
43	Giza 165	70.00	66.67	805.50	848.00	0.270	0.218
44	Sids 1	85.00	88.33	725.50	738.00	0.237	0.217
45	Sids 2	80.00	78.33	910.00	1003.50	0.202	0.237
46	Sids 3	88.33	85.00	1008.00	1008.00	0.219	0.219
47	Sakha 62	55.00	70.00	610.00	693.00	0.216	0.214
48	Sakha 69	60.00	68.33	709.50	888.50	0.217	0.217
49	Sohag 3	75.00	68.33	888.50	858.50	0.217	0.217
50	BanySwif 4	85.00	86.67	1016.50	1111.00	0.219	0.219
Mean		27.48	28.37	336.79	358.87	0.14	0.14
LSD 0.05		0.85		2.741		0.009	
LSD 0.01		1.112		3.609		0.005	

**Table 2:** ANOVA for leaf rust severity of 50 genotypes evaluated in Sids location during 2018/19 and 2019/20.

Mean Squares				
SOV	d.f	FRS	AUDPC	ACI
Replications	2	152.043 **	193.363	0.000 *
Treatments	99	2298.145 **	289,775.3 **	0.008 **
Genotypes (G)	49	4592.258 **	580,801.9 **	0.004 **
Years (Y)	1	39.603	45,534.72 **	0.002 **
G × Y	49	50.139 **	3733.289 **	0.002
Error	198	14.103	146.796	0.004

*, ** Significant at *p* ≤ 0.1, *p* ≤ 0.01, analysis of variance.

**Table 3:** Primer names, sequences, annealing temperature, product size, and references for *Lr* genes’ associated markers used in this study.

No.	Primer	Forward (5′-3′)	Reverse (5′-3′)	Ta (°C)	Product Size	Ref.
1	*Lr1*	GGGACAGAGACCTTGGTGGA	GACGATGATGATTTGCTGCTGG	65	760 b.p.	[35]
2	*Lr 9*	TCCTTTTATTCCGCACGCCGG	CCACACTACCCCAAAGAGACG	63	300 b.p.	[36]
3	*Lr10*	GAAGCCCTTCGTCTCATCTG	TTGATTCATTGCAGATGAGATCACG	61	282 b.p.	[37]
4	*Lr 13*	GTGCCTGTGCCATCGTC	CGAAAGTAACAGCGCAGTGA	58	130–280 b.p.	[38]
5	*Lr19*	CATCCTTGGGGACCTC	CCAGCTCGCATACATCCA	57	300 b.p.	[39]
6	*Lr20*	ACAGCGATGAAGCAATGAAA	GTCCAGTTGGTTGATGGAAT	55	300–430–542 b.p.	[40]
7	*Lr21*	CCAAAGAGCATCCATGGTGT	CGCTTTTACCGAGATTGGTC	Touchdown “56–65”	885 b.p.	[41]
8	*Lr22a*	AAGCTGACTTGTGCAGAGCT	AAACCCTTCTGCAACCCACA	Touchdown “56–65”	600 b.p.	[42]
9	*Lr 24*	TCTAGTCTGTACATGGGGGC	TGGCACATGAACTCCATACG	Touchdown “56–65”	110–199–280 b.p.	[43]
10	*Lr25*	CCACCCAGAGTATACCAGAG	CCACCCAGAGCTCATAGAA	Touchdown “56–65”	250 b.p.	[26]
11	*Lr26*	CATCCTTGGGGACCTC	CCAGCTCGCATACATCCA	Touchdown “56–65”	260 b.p.	[44]
12	*Lr27*	TTCCCATAACTAAAACCGCG	GGAACATCATTTCTGGACTTTG	57	160–180–200 b.p.	[45]
13	*Lr28*	CCCGGCATAAGTCTATGG TT	CAATGAATGAGATACGTGAA	Touchdown “56–65”	380 b.p.	[46]
14	*Lr29*	GTGACCTCAGGCAAT GCACACAGT	GTGACCTCAGAACCGATG TCCATC	Touchdown “56–65”	160 b.p.	[26]
15	*Lr32*	ATCGCCATCTCC TCT ACCA	GCGAACCCATGTGCTAAG	Touchdown “56–65”	240–273 b.p.	[43]
16	*Lr34*	GTGAAGCAGACCCAGAACAC	GACGGCTGCGACGTAGAG	Touchdown “56–65”	270 b.p	[47]
17	*Lr35*	AGAGAGAGTAGAAGAGCTGC	AGAGAGAGAGCATCCACC	Touchdown “56–65”	252 b.p.	[48]
18	*Lr36*	GCTGCATGAGCTCTGCAAT	TCTGTGAGGCATGACAGAA	55	480 b.p.	[49]
19	*Lr37*	AGGGGCTACTGACCAAGGCT	TGCAGCTACAGCAGTATGTACACAAAA	64	190–250 b.p.	[50]
20	*Lr39*	CCTGCTCTGCCCTAGATACG	ATGTGAATGTGATGCATGCA	Touchdown “56–65”	180–240–260 b.p.	[51]
21	*Lr46*	AGG GAAAAGACATCTTTTTTTTC	CGACCGACTTCGGGTTC	Touchdown “56–65”	335 b.p.	[52]
22	*Lr47*	AACTGGAAGCTGTACTCAGAG	GATGAACAATATGGGCAGG	Touchdown “56–65”	400–480 b.p.	[53]
23	*Lr48*	AATGGTTGTTCCCTCGACCT	CAAAAGGGAGAAAGGCGCAC	60	-	Unpublished
24	*Lr50*	GTCAGATAACGCCGTCCAAT	CTACGTGCACCACCATTTTG	60	-	[45]
25	*Lr52*	GGGTCTTCATCCGGAACTCT	CCATGATTTATAAATTCCACC	Touchdown “56–65”	140 b.p.	[45]
26	*Lr60*	ATTCACTTGCCCCTTTTAAACTCT	GAGCCGTAGGAAGGACATCTAGTG	Touchdown “56–65”	120 b.p.	[54]
27	*Lr63*	TGCACTTCCCACAAC ACATC	TTGCCACGTAGGTGATTTATGA	Touchdown “56–65”	180–200 b.p.	[55]
28	*Lr67*	GTGACCTCAGAACCGATGTCCATC	GCAAGGAAGAGTGTTCAGCC	Touchdown “56–65”	200–450 b.p.	[56]

**Table 4:** Presence and absence of leaf rust resistant genes/alleles within the genetic makeup of the 50 Egyptian wheat varieties.

	1	2	3	4	5	6	7	8	9	10	11	12	13	14	15	16	17	18	19	20	21	22	23	24	25	26	27	28	29	30	31	32	33	34	35	36	37	38	39	40	41	42	43	44	45	46	47	48	49	50
***Lr1***	−	+	−	−	−	−	−	−	+	+	+	−	+	−	−	−	−	+	−	−	+	+	−	−	−	−	−	−	−	−	−	−	−	−	−	−	−	−	+	+	−	−	−	−	−	−	−	−	−	−
***Lr10***	−	−	+	−	−	−	−	−	−	−	−	−	−	−	−	−	−	−	−	−	−	−	−	−	−	−	−	−	−	−	−	−	−	−	−	−	−	−	−	−	−	−	−	−	−	−	−	−	−	−
***Lr13−1***	−	−	−	−	−	−	−	−	+	+	−	−	−	−	−	−	−	−	−	−	−	−	−	−	−	−	−	−	−	−	−	−	−	−	−	−	−	−	−	−	−	−	−	−	−	−	−	−	−	−
***Lr13−2***	+	+	+	+	+	+	+	+	+	+	+	+	+	+	+	+	+	+	+	+	+	+	+	+	+	+	+	+	+	+	+	+	+	+	−	+	+	+	+	+	+	+	+	+	+	+	+	+	+	+
***Lr19***	+	+	+	+	+	+	+	+	+	+	+	+	+	+	+	+	+	+	+	+	+	+	+	+	+	+	+	+	+	+	+	+	+	−	+	+	+	+	+	+	+	+	+	+	+	+	+	+	+	+
***Lr20−1***	+	+	+	+	+	+	+	+	+	+	−	+	+	+	+	+	+	+	+	+	+	+	+	+	+	+	+	+	+	+	+	+	+	−	+	+	+	+	+	+	+	+	+	+	+	+	+	+	+	+
***Lr20−2***	−	+	−	−	−	+	−	−	−	−	−	+	+	+	+	+	+	+	+	+	+	+	+	+	+	−	+	−	+	+	+	+	+	−	−	+	+	+	+	+	+	+	+	+	+	+	+	+	+	+
***Lr20−3***	−	−	−	−	−	−	−	−	−	−	−	−	−	−	−	−	−	−	−	−	−	−	−	−	+	+	+	−	−	−	−	−	−	−	−	−	−	−	−	−	−	−	−	−	−	−	−	−	−	−
***Lr22a***	+	+	+	+	+	+	+	+	+	+	+	+	+	+	+	+	+	+	+	+	+	+	+	+	+	+	+	+	+	+	+	+	+	+	+	+	+	+	+	+	+	+	+	+	+	+	+	+	+	+
***Lr24−1***	+	+	+	−	+	+	+	+	−	+	−	+	+	−	+	+	+	−	+	+	+	−	+	−	−	−	−	+	−	−	+	+	−	+	+	+	+	−	+	−	+	+	+	+	+	+	+	−	−	+
***Lr24−2***	−	−	−	−	−	−	−	−	−	−	−	−	−	−	−	+	−	−	−	−	−	−	−	−	−	+	−	−	−	−	+	−	−	−	−	+	−	−	−	+	−	−	+	−	−	−	−	−	−	+
***Lr24−3***	−	−	−	−	−	−	−	−	−	−	−	−	−	+	−	−	−	−	−	−	−	−	−	−	−	−	−	−	−	−	+	−	−	−	+	+	+	+	−	+	−	+	+	+	+	−	−	+	−	−
***Lr25***	−	+	+	−	−	−	−	−	−	−	−	−	−	−	−	−	−	+	−	−	−	−	−	−	−	−	−	−	−	−	−	−	−	−	−	−	−	−	−	−	−	−	−	−	−	−	−	−	−	−
***Lr27−1***	−	−	+	+	−	+	+	+	+	+	+	−	+	−	−	−	−	−	−	+	+	−	−	+	+	+	+	−	−	−	−	−	−	−	+	−	+	−	+	−	−	−	−	−	−	−	−	−	−	−
***Lr27−2***	+	+	−	−	+	−	+	−	−	−	−	+	−	+	+	+	+	+	+	−	−	+	+	−	−	−	−	+	+	+	−	−	−	+	−	+	−	+	−	+	+	−	−	−	−	−	−	−	+	−
***Lr27−3***	−	−	−	+	−	−	−	−	−	−	−	−	−	−	−	−	−	−	−	−	−	−	−	−	−	−	−	−	−	−	+	+	+	+	−	−	−	−	−	−	−	+	+	+	+	+	+	+	−	+
***Lr28***	+	+	+	+	+	+	+	+	+	+	+	+	+	+	+	+	+	+	+	+	+	+	+	+	+	+	+	+	+	+	+	+	+	+	+	+	+	+	+	+	+	+	+	+	+	+	+	+	+	+
***Lr29***	+	+	+	+	+	+	+	+	+	+	+	+	+	+	+	+	+	+	+	+	+	+	+	+	+	+	+	+	+	+	+	+	+	+	+	+	+	+	+	+	+	+	+	+	+	+	+	+	+	+
***Lr32***	+	+	+	+	+	+	+	+	+	+	+	+	+	+	+	+	+	+	+	+	+	+	+	+	+	+	+	+	+	+	+	+	+	+	+	+	+	+	+	+	+	+	+	+	+	+	+	+	+	+
***Lr34***	+	+	+	+	+	+	+	+	+	+	+	+	+	+	+	+	+	+	+	+	+	+	+	+	+	+	+	+	+	+	+	+	+	+	+	+	+	+	+	+	+	+	+	+	+	+	+	+	+	+
***Lr36***	+	+	+	+	+	+	+	+	+	+	+	+	+	+	+	+	+	+	+	+	+	+	+	+	+	+	+	+	+	+	+	+	+	−	+	+	+	+	+	+	+	+	+	+	+	+	+	+	+	+
***Lr37−1***	+	+	−	−	−	−	−	−	−	+	−	−	+	+	−	−	−	−	−	+	+	−	−	+	−	+	−	+	+	−	−	−	+	−	+	−	−	−	−	−	+	−	−	−	+	+	−	−	−	+
***Lr37−2***	−	+	−	+	+	+	+	+	+	+	−	+	+	+	−	−	+	−	−	−	+	−	+	+	−	−	+	+	+	−	+	+	−	−	−	−	+	+	−	+	+	−	+	+	−	+	+	+	−	+
***Lr39−1***	−	−	+	−	−	−	+	+	+	+	−	−	−	+	−	−	−	−	−	−	−	−	−	−	+	−	−	−	−	−	−	−	−	−	−	−	−	−	−	−	−	−	−	−	−	−	−	−	−	−
***Lr39−2***	+	+	+	+	+	+	+	+	+	+	+	−	+	+	+	−	−	+	+	+	+	+	+	+	+	+	+	+	−	−	+	+	+	−	+	−	−	−	+	+	−	−	−	−	−	−	−	−	+	−
***Lr39−3***	−	−	−	−	−	−	−	−	−	−	−	+	−	−	−	+	+	−	−	−	−	−	−	−	−	−	−	−	+	+	−	−	−	+	−	+	+	+	−	−	+	−	−	−	−	−	−	−	−	−
***Lr47−1***	+	+	+	+	+	+	+	+	+	+	+	+	+	+	+	+	+	+	+	+	+	+	+	+	+	+	+	+	+	+	+	+	+	+	+	+	+	+	+	+	+	+	+	+	+	+	+	+	+	+
***Lr47−2***	+	+	+	+	+	+	+	+	+	+	+	+	+	+	+	+	+	+	+	+	+	+	+	+	+	+	+	+	+	−	+	+	+	−	−	+	+	+	+	+	+	+	+	+	+	+	+	+	+	+
***Lr52***	+	+	+	+	+	+	+	+	+	+	+	+	−	−	+	−	−	−	−	+	+	+	−	−	+	−	+	−	−	−	+	−	−	+	+	+	+	+	+	+	+	−	−	−	−	−	+	−	+	−
***Lr60***	+	+	+	+	+	+	+	+	+	+	+	+	+	+	+	+	+	+	+	+	+	+	+	+	+	+	+	+	+	+	+	+	+	+	+	+	+	+	+	+	+	+	+	+	−	+	+	+	+	−
***Lr63−1***	+	−	+	+	+	+	−	+	−	−	−	−	−	+	+	+	+	−	+	−	−	+	+	−	−	−	−	+	+	+	−	−	−	−	+	−	−	−	+	+	−	−	−	−	−	−	−	−	+	−
***Lr63−2***	−	+	−	−	−	−	+	−	+	+	+	+	+	−	−	−	−	+	−	−	−	−	−	+	−	+	+	−	−	−	+	+	+	−	−	+	+	+	−	−	+	+	+	+	+	+	+	+	−	+
***Lr67−1***	+	+	+	+	+	+	+	−	+	+	+	+	+	+	+	+	+	+	+	+	+	+	+	+	−	+	+	−	−	+	+	+	+	+	+	+	+	+	+	+	+	+	+	+	−	+	+	+	+	+
***Lr67−2***	−	−	−	+	−	−	−	−	−	−	−	+	−	−	−	−	−	−	−	−	−	−	−	−	−	−	−	−	−	−	−	−	−	−	−	−	−	−	−	−	−	+	−	−	−	−	−	−	+	+

**Table 5:** Primers, number of monomorphic bands, number of polymorphic bands, percentage of polymorphism, and marker efficiency parameters of leaf rust resistance genes.

Primer	NMB *	NPB **	% ^§^ Polymorph.	H	PIC	E	H. av	MI	D	R
*Lr1*	0	1	100%	0.32	0.269	0.2	0.0064	0.0013	0.9633	0.4
*Lr10*	0	1	100%	0.039	0.03843168	0.02	0.000784	0.0000157	1	0.04
*Lr13*	0	2	100%	0.499	0.37489998	1.02	0.004998	0.005098	0.7424242	0.12
*Lr19*	0	1	100%	0.039	0.03843168	0.98	0.000784	0.0007683	0.04	0.04
*Lr20*	0	3	100%	0.485	0.367376055	1.76	0.003233	0.0056904	0.6574497	0.72
*Lr22a*	1	0	0%	0	0	1	0	0	0	0
*Lr24*	0	3	100%	0.453	0.350383	1.04	0.00302	0.003141	0.881342	1.44
*Lr25*	0	1	100%	0.113	0.106438	0.06	0.002256	0.000135	0.997551	0.12
*Lr27*	0	3	100%	0.457	0.352563433	1.06	0.003047	0.0032293	0.8766890	2.12
*Lr28*	1	0	0%	0	0	1	0	0	0	0
*Lr29*	1	0	0%	0	0	1	0	0	0	0
*Lr32*	1	0	0%	0	0	1	0	0	0	0
*Lr34*	1	0	0%	0	0	1	0	0	0	0
*Lr36*	0	1	100%	0.039	0.038432	0.98	0.000784	0.000768	0.04	0.04
*Lr37*	0	2	100%	0.498	0.374098	0.94	0.004982	0.004683	0.781616	1.48
*Lr39*	0	2	100%	0.44	0.34315	0.98	0.002933	0.002874	0.894765	1.4
*Lr47*	1	1	50%	0.058	0.056506	1.94	0.000582	0.001129	0.059394	0.12
*Lr52*	0	1	100%	0.487	0.368518	0.58	0.009744	0.005652	0.668571	0.84
*Lr60*	0	1	100%	0.077	0.073851	0.96	0.001536	0.001475	0.079184	0.08
*Lr63*	0	2	100%	0.497	0.373395	0.92	0.004968	0.004571	0.790909	1.76
*Lr67*	0	2	100%	0.5	0.375	1	0.005	0.005	0.752525	0.4

* Number of monomorphic bands; ** number of polymorphic bands; ^§^ percentage of polymorphism.

**Table 6:** Pedigree and year of release of the wheat varieties under study.

Code	Verity	Pedigree	Year	Yield/Hectare (T/H)
1	BanySwif 1	JO’’S’’/AA’’S’’//FG’’S’’	1987	6.3
2	BanySwif 4	AUSL/5/CANDO/4/BY*2/TAC//II27655/3/TME//ZB/W*2.ICD88-1120-ABL-0TR-1BR-0TR-6AP-0AP-OSD	2007	6.3
3	BanySwif 5	DIPPERZ/BUSHEN3.CDSS92B128-1M-0Y-3B-0Y-0SD.	2007	6.4
4	BanySwif 6	BOOMER-21/BUSCA-3.CDSS95Y01185-8Y-OM-0Y-0B-1Y-0B0SD	2010	6.5
5	BanySwif 7	CBC509CHILE//sooty_9/RASCON_37/9/USDA595/3/D67.3/RABI//CRA/4/ALO/5/HUI/YAV_1/6/ARDENTE/7/HUI/YAV79/8/POD_9CDSS02Y01233T-0OTOPB-0Y-0M-26Y-0Y-0SD	2017	6.8
6	Gemmeiza 1	Maya74/0n//1160-147/3/Bb/1991 Gall/4/chat “S”CM58924-IGM-OGM	1991	5.83
7	Gemmeiza 11	BOW’’S’’/KVZ’’S’’//7C/SERI82/3/GIZA168/SKHA61.	2011	6.59
8	Gemmeiza 12	OTUS/3/SARA/THB//VEE.CCMSS97Y00227S-5Y-010M-010Y-010M-2Y-1M-0Y-0GM	2018	6.65
9	Gemmeiza 3	Bb/7C*2//Y50/KaL*3//Sakha8/4/Prv/WW/5/3/Bg/”S” ONCGM.4024 -IGM-13GM-2GM-0GM.	1997	6.08
10	Gemmeiza 5	Vee”S”/SWM6525CGM.4017-1GM-6GM-3GM-0GM.	1998	6.08
11	Gemmeiza 7	CMH74A.630/5X//Seri 82/3 Agent CGM.4611-2GM.-3GM.-1GM.-0CM.	1999	6.55
12	Gemmeiza 9	Ald”S”/Huas//CMH74A.630/SxCGM4583-5GM-1GM-0GM.	1999	6.55
13	Giza 139	HINDI90/KENYA256G.	1947	2.14
14	Giza 144	REGENT/G.139	1958	2.61
15	Giza 155	REGENT/2∗GIZA139//MICADET/2∗HIND162	1968	3.08
16	Giza 156	RIO NEGRO/2∗MENATANE//KENYA/3∗2GIZA135/LTNE950	1972	3.08
17	Giza 157	GIZA155//PIT62/*LR*64/3/TZPP/KNOTT	1977	4.99
18	Giza 160	Chenab 70/Giza 155	1982	5
19	Giza 162	Vcm//Cno 67/7C/3/Kal/Bb CM8399-D-4M-3Y-1M-1Y-1M-0Y	1987	5.62
20	Giza 163	T. aestivum/Bon//Cno/7C CM33009-F-15M-4Y-2M-1M-1M-1Y-0M	1987	5.62
21	Giza 164	KVZ/Buha “s”//Kal/Bb CM33027-F-15M-500y-0M	1987	5.62
22	Giza 165	0MCno/Mfd//Mon “S” CM43339-C-1Y-1M-2Y-1M-2Y-0B	1991	5.83
23	Giza 167	Au/UP301//G11/SX/Pew”S”/4/Mai”S”/May”S”//Pew”S” CM67245-C-1M-2Y-1M-7Y-1M-0Y	1995	6.08
24	Giza 168	Au/UP301//G11/SX/Pew”S”/4/Mai”S”/May”S”//Pew”S” CM67245-C-1M-2Y-1M-7Y-1M-0Y	1995	6.55
25	Giza 171	Sakha 93/Gemmeiza 9 S.6-1GZ-4GZ-1GZ-2GZ-0S	2013	6.61
26	Hendy 62	selectable from local cultivars	1926	1.56
27	Mabrok	GIZA7/BALADI42.	1921	1.73
28	Misr 2	SKAUZ/BAV92. CMSS96M03611S-1M-010SY010M-010SY-8M-0Y-0S.	2011	6.4
29	Montana	selectable from local cultivars	-	2.4
30	Nubaria 1	OASIS/5*BOR95/5/CNDO/R143//ENTE/MEX175/3/CNDO/R143	-	6.02
31	Romana	selectable from local cultivars	-	2.3
32	Sakha 61	Inia–RL4220//7C/YR”S” CM15430-25-55-0S-OS	1980	5
33	Sakha 62	GIZA7/BALADI42.	1980	5
34	Sakha 69	Inia–RL4220’7C/YR”S”CM15430- 25 -65-0S-0S	1980	5
35	Sakha 8	Indus66*Norteno”S”-PK348	1976	5
36	Sakha 88	KVZ/TI/3/MAYA74 “S”//BB/TNTA	1985	6.1
37	Sakha 92	NAPO63/TNT1A66//WERN “S”	1987	5.62
38	Sakha 94	Opata/Rayon//Kauz CMBW9043180-OTOPM-3Y-010M-010M-010Y-10M-015Y-0Y	2004	6.55
39	Sakha 95	POSTOR//SITE/MO/3/CHEN/AEGILOPS/SQUARROSA(TAUS)	2018	6.55
40	Sids 1	HD2172/Pavon “S”//1158.57/Maya74 “S” SD46-4Sd-2SD-1SD-0SD	1996	6.08
41	Sids 14	KAUZ”S”//TSI/SNB”S”. ICW94-0375-4AP-2AP-030AP-0APS-3AP.	2014	6.65
42	Sids 2	HD2206/HORK “S”/3/NAPO63/NAPO63/INIA66//WREN “S”	1996	6.08
43	Sids 3	SAKA69/GIZA155	1996	6.08
44	Sids 5	MAYA “S”/MON “S”/MON “S”//CMH74.592/3/GIZA157∗2	1996	6.09
45	Sids 6	Maya”s”/Mon “s”/CMH74.A592/3/Sakha 8*2SD10002-4SD-3SD- 1SD -0SD	1996	6.08
46	Sids 7	Maya “S”/Mon “S”//CMH74A.592/3/Sakha8∗2	1996	6.03
47	Sids 8	Maya “S” Mon “S”/CMH74. A592/3/Sakha 8*2SD10002-14SD-3SD-1SD-0SD.	1996	6.08
48	Sohag 3	MIEX’’ S’’/M G HA/51792//D URUM6.	1991	6.3
49	Sohag 4	Ajaia-16//Hora/Jor/3/Gan/4/Zar/5/Souk-7/6/Stot//Altar84/aLdCDSS99B00778S-0TPY-0M-0Y-129Y-0M-0Y-1B-0SH	1998	6.4
50	Sohag 5	Ajaia-16//Hora/Jro/3/Gan/4/Zar/5/Suok-7/6/Stot//Altar84/AldCDSS99B00778S-OTOPY-0M-0Y-129Y-0M-0Y-1B-0SH	2016	6.6

## Data Availability

Not applicable.
